# Bariatric Surgery With Roux-En-Y Gastric Bypass or Sleeve Gastrectomy for Treatment of Obesity and Comorbidities: Current Evidence and Practice

**DOI:** 10.7759/cureus.25762

**Published:** 2022-06-08

**Authors:** Daniel Chacon, Timothy Bernardino, Feargal Geraghty, Astrid Carrion Rodriguez, Brian Fiani, Asadulla Chadhaury, Muller Pierre-Louis

**Affiliations:** 1 School of Medicine, Ross University, Bridgetown, BRB; 2 Trauma, Hospital Corporation of America (HCA) Kendall Regional Medical Center, Miami, USA; 3 Department of Internal Medicine, Cleveland Clinic Florida, Weston, USA; 4 Neurosurgery, Weill Cornell Medical Center/New-York Presbyterian Hospital, New York, USA; 5 Department of General and Bariatric Surgery, Cleveland Clinic Martin North Hospital, Stuart, USA

**Keywords:** medical comorbidities, sleeve gastrectomy, gastric bypass, bariatric surgery, type ii diabetes, obesity

## Abstract

Background: With the growing prevalence of obesity in the global population, alternative measures for weight loss and treatment of comorbidities must be considered due to the increasing difficulty of conservative management alone. Here we discuss the benefits of bariatric surgery on weight loss as well comorbidities that are present in a majority of obese patients.

Methods: In this review, we discuss the current practice and evidence of bariatric surgery as it pertains to weight loss and the beneficial effect on comorbidities commonly present in obesity.

Results: Our review found that bariatric surgery with either the roux-en-y gastric bypass or laparoscopic sleeve gastrectomy can result in weight loss of up to 80% of excess weight. We also found that bariatric surgery has a profound effect on multiple comorbidities such as type 2 diabetes mellitus, hypertension, and hyperlipidemia through remission of the disease.

Conclusion: Bariatric surgery serves as an efficacious alternative for treatment of obesity and comorbidities.

## Introduction and background

The rapid progression of obesity in the population has been a crippling challenge faced by the healthcare system for decades. In fact, since 1975, the prevalence of obesity worldwide has nearly tripled [1]. In a recent poll by the World Health Organization (WHO) in 2016, 1.9 billion and 650 million individuals over the age of 18 were considered overweight and obese, respectively [1]. Dating back as far as the 1920s, medical management was largely accepted as the modality of choice for addressing obesity and aiding patients in weight loss. The multifaceted approach focused on low-calorie diets, exercise, anorectic drugs, and behavioral therapy, which had little to no success in long-term outcomes for patients [2]. Over the ensuing six decades, the failure of medical management and the success of surgical intervention led to the substantial emergence of novel practices and procedures in the treatment of obesity and its associated comorbidities.

Much like the original medical approach to weight loss, the current philosophy of bariatric surgery involves a multidisciplinary team working in unison to ensure the attainment and maintenance of weight loss. Long before entering an operating room, the process begins with extensive education on nutrition, lifestyle modifications, and addressing psychological deterrents necessary for an obese patient to undergo successful bariatric surgery. In conjunction with these behavioral modifications, the surgical procedures performed can be classified into one of three categories: volume restrictive, nutrient malabsorptive, or a combination of both. These surgical procedures ultimately affect satiety, absorption, and neurohormonal effects on the regulation of energy expenditure and hunger control. 

Volume restrictive procedures limit caloric intake via reduction of the gastric reservoir capacity via resection, bypass, or creation of a proximal gastric outlet. Within this surgical category, two purely restrictive procedures include vertical banded gastroplasty (VBG) and laparoscopic adjustable gastric banding (LAGB). Both limit the volume of solid food able to be consumed solely due to the constrained stomach size, leaving the absorptive capabilities of the small intestine intact. However, these two procedures have largely fallen out of favor and have been replaced by the most frequently performed bariatric procedure in the USA and the world: the laparoscopic sleeve gastrectomy (LSG), accounting for 59.4% of all bariatric procedures performed in 2019 [3,4].

Malabsorptive procedures decrease the effectiveness of nutrient absorption by decreasing the length of small bowel capable of food breakdown by one of two mechanisms. These mechanisms are either via bypass of the small bowel absorptive surface area, or diversion of the biliopancreatic digestive secretions that facilitate absorption. Jejunoileal bypass (JIB) and biliopancreatic diversion (BPD) are examples of purely malabsorptive procedures which result profound weight loss. However, due to their substantial metabolic complications related to malabsorption, utilization of these surgical interventions has been overtaken by procedures that adopt qualities of both restrictive and malabsorptive approaches [3]. Procedures that utilize a combination of both restrictive and malabsorptive properties include the well-established Roux-en-Y gastric bypass (RYGB) and biliopancreatic diversion with duodenal switch (BPD/DS). Of these two, RYGB is second only to the LSG as the most commonly employed bariatric surgery in the world, accounting for 17.8% of all bariatric procedures performed in 2019 [3,4].

Here we will present and discuss the current practice of bariatric surgery, highlighting the surgical procedure and pre-operative requirements for candidacy. Followed by an extensive review and presentation of current literature reporting on the post-surgical outcomes as it pertains to efficacy on weight loss and beneficial effects on comorbidities often present in obesity.

Methods

Our literature search and selection criteria followed the PRISMA guidelines. We used a database of published open access articles filtered by keywords including “post-surgical outcomes”, “bariatric surgery”, “comorbidities”, “roux-en-Y gastric bypass”, and “laparoscopic sleeve gastrectomy”, resulting in 54 potential articles to include in our review.

Inclusion criteria for our review consisted of articles reporting weight loss at multiple periods, postoperatively, and the effect of surgery on comorbidities, including type 2 diabetes mellitus, hypertension, and hyperlipidemia. Articles reporting on only one period were not excluded; however, the minimum period post-operatively was to be six months. Exclusion criteria consisted of articles of non-English language, non-human subjects, articles with less than six months postoperative follow-up, and articles including values on surgical techniques other than Roux-en-Y gastric bypass (RYBG) and the LSG were not included in our review. A final count of articles used in our review totaled to 32.

Surgical procedure

The American Society of Metabolic and Bariatric Surgery (ASMBS) currently endorses seven bariatric procedures that health centers accredited by the American College of Surgeons Metabolic and Bariatric Surgery Accreditation and Quality Improvement Program (MBSAQIP) may perform [5]. Here we will review the general steps involved in the two most common surgeries- the laparoscopic sleeve gastrectomy (LSG) and the Roux-en-Y gastric bypass (RYGB). Note: certain steps involving patient positioning, prepping, draping, and closing have been omitted for convenience purposes.

**Figure 1:**
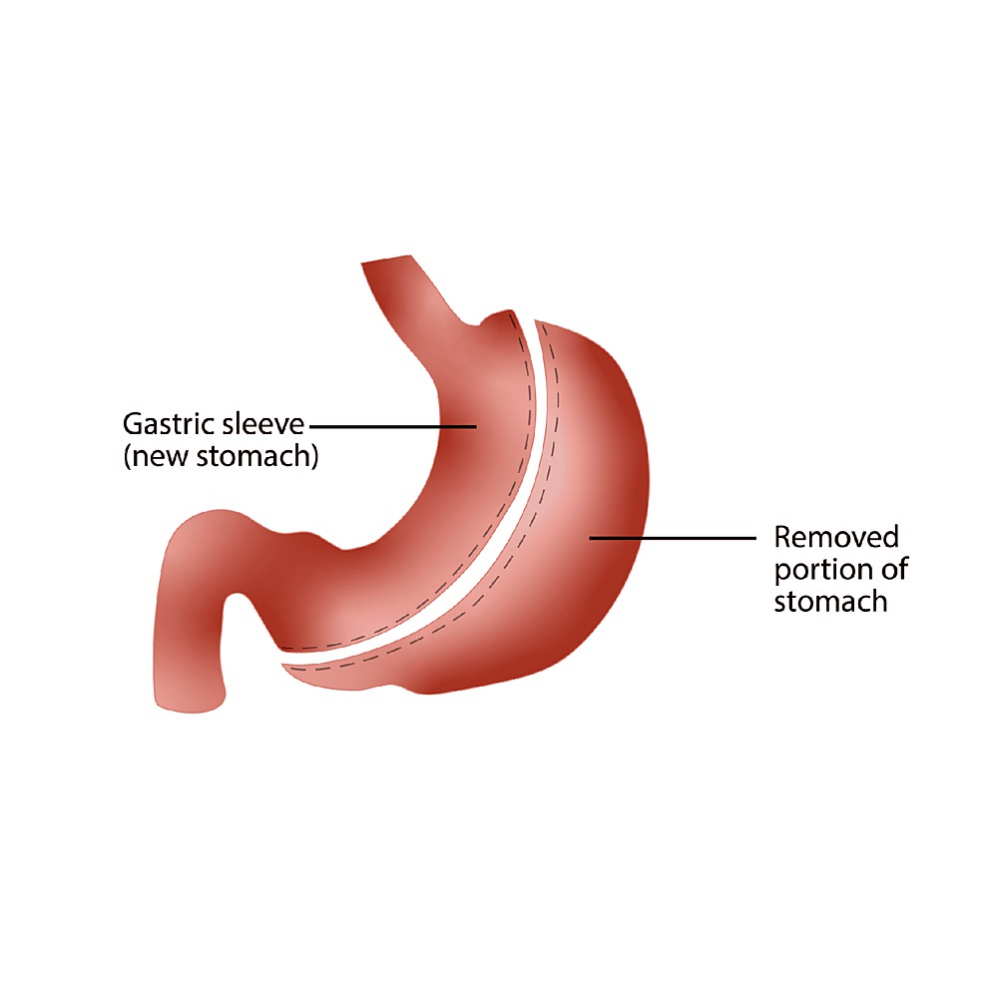
Anatomical view of the sleeve Gastrectomy. (Original Artwork by Rogelio Avila)

The restrictive sleeve gastrectomy was first performed laparoscopically in 1999 and has since become the most common bariatric surgery performed in the world, overtaking RYGB in 2015 [3,6]. The procedure is initiated with the insertion of a trocar into the abdominal cavity just superior to the umbilicus utilizing a Hassan or Optiview technique. Following appropriate insufflation, three to four additional trocars are introduced under direct visualization with a 30-degree laparoscope. A liver retractor is often utilized for improved visualization and avoidance of injury to hepatobiliary structures. Dissection and mobilization of the greater curvature and lesser curvature (or lesser and greater curvatures) of the stomach is then achieved with the aid of laparoscopic energy devices. With the stomach completely mobilized, a 32 to 40 French bougie is placed in the lesser curve segment prior to resection to maintain adequate lumen and achieve a standardized size of gastric remnant. With the bougie properly placed, an endoscopic GIA stapler is introduced, stapling the stomach at a point proximal to the pylorus for preservation of the antrum and to allow for maintenance of gastric emptying. The staple lines are sequentially fired along the bougie toward the angle of His and divide the fundus just lateral to the esophagus. Staple lines are often reinforced with sutures ensuring less chance of staple line leaks. Roughly 80% of the stomach is removed, with the fundus making up the largest portion of the excised specimen. The procedure is non-adjustable and non-reversible. The duration of the procedure is between 30 and 90 minutes, with an average 1-2 day stay in the hospital post-procedurally [3,6]. A view of the surgical procedure is shown on Figure 1.

**Figure 2:**
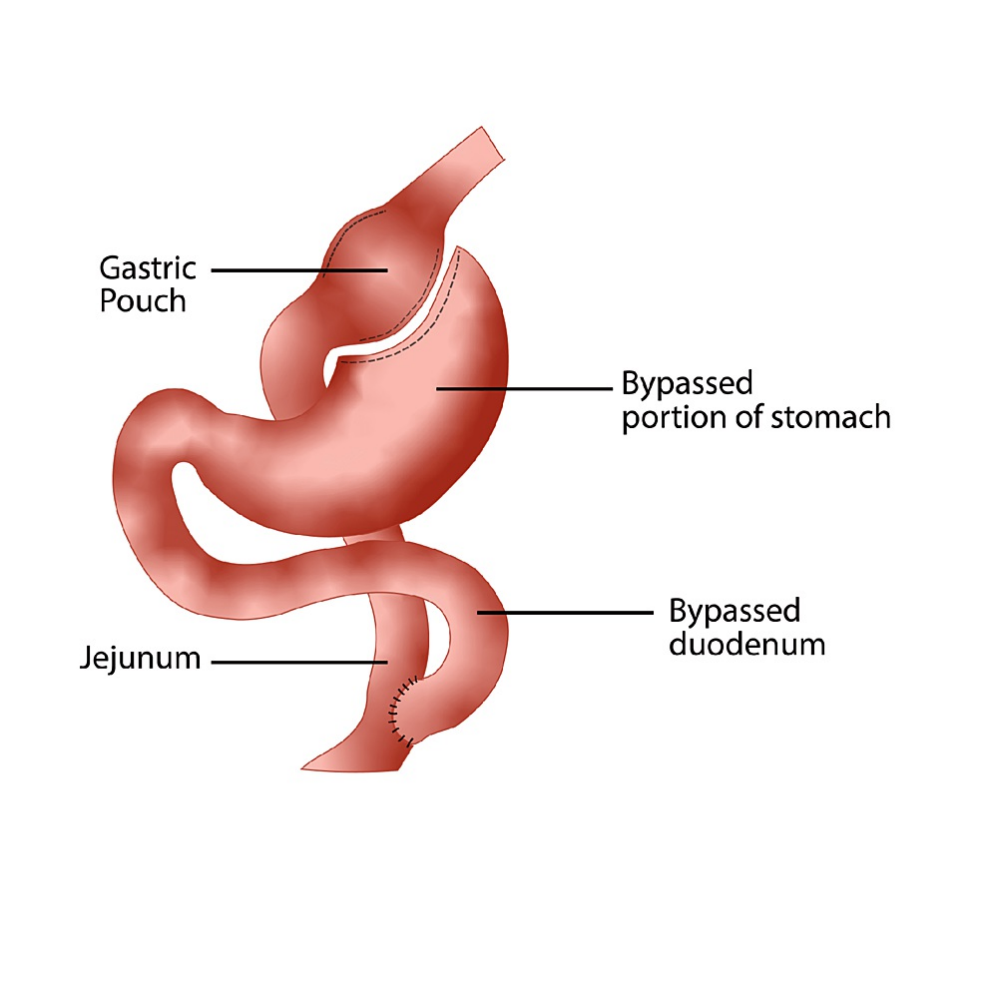
Anatomical View of the Roux-En-Y Gastric Bypass (Original Artwork by Rogelio Avila)

The restrictive-malabsorptive Roux-en-Y gastric bypass (RYGB) procedure was first performed laparoscopically in 1994 [7]. The process begins in similar fashion to the sleeve gastrectomy, with entry into the abdominal cavity via insertion of a trocar superior to the umbilicus utilizing the Hassan or Optiview technique. Following appropriate insufflation, an additional four to five trocars are inserted under direct visualization with a 30-degree laparoscope. Once again, a retractor is often employed to increase gastric exposure and protect the liver and associated structures. After proper dissection and mobilization, the next step involves creation of the gastric pouch. The pouch creation involves multiple GIA linear staple firing to the proximal stomach resulting in a closed, 20-30 cc pouch. The resultant large residual stomach is now completely excluded from food entry via the esophagus. Next is the creation of the biliopancreatic (BP) -or afferent limb- which consists of the duodenum and proximal jejunum remaining in continuity with the remnant stomach. In a standard gastric bypass, approximately 40-50 cm is measured starting at the ligament of Treitz and divided using a stapling device to create the BP limb. The roux, or efferent, limb is subsequently created. This limb which consists of mobilizing and measuring 75 to 150 cm of jejunum distal to the division point. The first anastomosis follows with the creation of the jejunojejunostomy. The BP limb is anastomosed to the distal segment of jejunum to create a side-to-side jejunojejunostomy, or the JJ anastomosis. The roux limb of the jejunum can then be brought up either in an antecolic-antegastric or a retrocolic-retrogastric orientation. A side-to-side gastrojejunostomy is created next by anastomosis of the proximal end of the mobilized jejunum to the gastric pouch. As in the LSG, staple lines involved in the anastomoses are reinforced with sutures to minimize the risk of staple leaks. The procedure takes 90-150 mins with an average two-to-four-day hospital stay. Most centers employ a post-operative leak test within 24-36 hours via an upper gastrointestinal series to rule out the presence of anastomotic leaks prior to hospital discharge [3,7]. A view of the surgical procedure is shown in Figure 2.

Clinical indications and preoperative evaluation for bariatric surgery

Multiple steps are required for a patient to be considered a candidate for bariatric surgery. This process begins with satisfying inclusion criteria related to body mass index (BMI) and obesity-associated comorbidities. According to the ASMBS and Society of American Gastrointestinal and Endoscopic Surgeons (SAGES) guidelines for bariatric surgery, the indications for weight loss surgery are as follows [8,9]: 1) BMI ≥ 40 kg/m2 with no comorbid conditions. 2) BMI of 35-39.9 kg/m2 with at least one serious comorbidity, including but not limited to type-2 diabetes mellitus, hypertension, obstructive sleep apnea (OSA), hyperlipidemia, obesity hypoventilation syndrome (OHS), Pickwickian syndrome, nonalcoholic fatty liver disease, pseudotumor cerebri, or severe limitations on the quality of life-related to weight. 3) BMI of 30-34.9 kg/m2 with metabolic syndrome or diabetes mellitus that is uncontrolled with medical therapy.

It is also important to note that candidacy for bariatric surgery additionally depends on fulfilling the above criteria and those criteria decided per the surgical institution. This often entails the documented failure of previous non-surgical attempts at weight reduction and/or participation in a pre-operative guided weight loss program with exercise and lifestyle modifications [10]. In addition, certain commitments may also be required by surgical programs to ensure the patient will abide by the postoperative nutritional restrictions required to successfully achieve the desired weight loss. These commitments vary amongst the various surgical institutions but may include attending a mandatory information seminar on bariatric surgery, expectations of patient adherence to postoperative care, including follow-up visits with the surgeon and other members of the care team.

As previously mentioned, the extensive pre-operative assessment encompasses a multidisciplinary team, often involving dieticians, psychologists, endocrinologists, nurses, anesthesiologists, and cardiologists working in conjunction with the surgical team to ensure a positive postoperative outcome [8]. Although the preoperative workup and evaluation require tailoring unique to each patient, the following interventions have become a mainstay in evaluating the comorbidities, expectations, and medical and psychological concerns the patient may have.

A psychological evaluation is one of the first steps in preoperative evaluation. This involves screening for a history of mental disorder, depression, eating disorders, prior weight loss attempts, compliance with therapy, and substance misuse. Centers may require documented smoking abstinence, as well as referral for rehabilitation and detoxification if alcohol dependence is identified, with subsequent documentation of abstinence before surgery [8].

Following psychiatric evaluation, patients undergo a nutritional assessment by a licensed dietician or nutritionist. This primarily involves patient education and guidance towards long-term dietary modifications required postoperatively. Importantly, discussion regarding patient expectations on the amount of weight loss following the procedure should be addressed, as well as weight loss maintenance strategies. The licensed dietician will also contribute to the glycemic control in diabetic patients as well as pre- and post-operative monitoring of serum vitamin levels that may require supplementation in patients undergoing malabsorptive procedures [8]. Although the key mechanism responsible for the weight loss will be the surgical intervention, a documented weight loss plan is of utmost importance. The plan should include a guided exercise program along with a structured diet to be applied prior to the surgery. Failure of program adherence and diet preoperatively may be a useful predictor of how reliable a patient will be with implementing lifestyle modifications needed in the postoperative period [8].

Required for all surgical procedures, the next preoperative evaluation involves medical risk assessment. The patient should undergo a detailed history and physical with careful attention to uncontrolled comorbid conditions, as well as screening for undiagnosed obesity-related comorbidities. Most importantly, an evaluation of cardiopulmonary health should be assessed with an EKG and chest radiography. If indicated based on a patient’s past medical history, cardiac risk assessment may additionally be required. Laboratory workup often includes a complete blood count, complete metabolic panel, lipid panel, thyroid function panel, serum iron, vitamin B-12, folate levels, blood type and screen, and hemoglobin A1C [8].

Though likely to vary amongst surgeons and institutions, a final step of preoperative management is preoperative imaging. Based on careful consideration of past surgical and medical history as well as the particular surgical intervention planned, certain imaging modalities may be indicated. For example, in a patient planned to undergo RYGB, an abdominal ultrasound may be performed to rule out cholelithiasis, a condition with high postoperative prevalence in this select patient population. An upper endoscopy (EGD) is also indicated in the bypass preoperative assessment to identify the presence of any gastric or duodenal pathologies that would require future surveillance or intervention given access to the bypassed foregut postoperatively structures would prove challenging. Likewise, in patients undergoing LSG, an EGD is often ordered to evaluate for evidence of gastroesophageal reflux disease (GERD) or esophagitis, both of which tend to worsen in this patient population [8].

Contraindications

In view of the aforementioned indications for surgery, it is important to discuss the multiple contraindications and exclusion criteria that exist. Several medical and psychiatric conditions that exclude patients from qualifying as bariatric surgical candidates are listed below [3,9-11]: 1) Untreated major depression or psychosis. 2) Uncontrolled and untreated eating disorders (e.g., Bulimia). 3) Current drug or alcohol abuse. 4) Severe cardiac disease with prohibitive anesthetic risk (ASA score of IV). 5) Severe coagulopathy. 6) Portal hypertension. 7) Reversible endocrine or other disorders that can cause obesity. 8) Inability to comply with nutritional requirements, including lifelong vitamin replacement. 9) Lack of comprehension of risks, benefits, outcomes, alternatives, and lifestyle changes. 10) RYGB specific: Crohn’s disease (relative contraindication). 11) LSG specific: Severe esophagitis/Barrett’s esophagus (relative contraindication).

In addition, there does exist some debate on relative contraindications related to the age of possible candidates, namely those aged >65 years old and <18 years old. In the pediatric and adolescent populations, bariatric surgery is becoming more common. Notably, bariatric surgery was recently endorsed by the American Academy of Pediatrics, with the recommendation that only high-quality programs offering both pediatric and family-specific care be permitted to perform surgery on this young population [12]. With regards to patients >65 years, the decision is ultimately based on the overall health of the patient, specifically based on the presence of cardiopulmonary disease that increases individual American Society of Anesthesiology (ASA) scores and decreases tolerability to surgical procedures [13]. Lastly, it was previously established that bariatric procedures should not be offered as a solution for greater control of lipids and glucose or as a method to reduce cardiovascular risk independently of the BMI parameters [14]. However, when reviewing outcomes during post-operative evaluation, bariatric surgery has been shown to improve such parameters.

## Review

Post-surgical outcomes

Weight Loss

Bariatric surgery has shown to be an excellent option for weight loss for individuals with obesity secondary to failure of conservative management. Currently, both RYGB and LSG are favorable procedures. Obesity is defined based on body mass index (BMI), a value calculated by the ratio of an individual’s weight in kilograms (kg) to height in meters squared (m2) [15], with BMI greater or equal to 30 kg/m2 qualifying as obesity. Measurable parameters include excess weight (EW), calculated as the difference between pre-operative weight and body weight at ideal BMI [16]. The Center for Disease Control and Prevention (CDC) defines ideal body weight as a BMI of 18.5-24.9; however, most bariatric studies use an ideal BMI between 23-25 [15-17]. Percentage of excess weight loss (% EWL), calculated as the total weight loss divided by the difference between pre-operative body weight and body weight at ideal BMI [17], is often measured as well. Patients are asked to attend clinic follow-up visits for the continuance of weight loss measurement and post-operative assessments. Subsequent visits typically occur during the two-month to two-year postoperative period; however, some studies have followed patients for up to five years [16-22]. 

Due to the changes in absorption and decreased reservoir capacity of the stomach, weight loss has been the most prominent outcome. In multiple studies, both prospective and retrospective observational studies, patients were followed postoperatively with weight loss recorded throughout various time intervals [16-22]. In all studies conducting a comparative analysis of the two procedures, RYGB was shown to have a higher %EWL at all periods, including two months, six months, and annually during years one to five postoperatively [16-22]. However, both the RYGB and SG show significant weight loss at all periods postoperatively. Notably, a few reports have shown %EWL reach as high as 80% and consistent weight loss for up to five years [16,18-21]. Further suggests that bariatric surgery remains an effective option for weight loss in obesity. Details of %EWL for both RYGB and SG for the studies are listed in Table 1.

**Table 1:** Review of Percentage Excess Weight Loss

	Procedure Performed	Post-Operative Follow Up and Percentage of Excess Weight Loss					
		6 Months	1 Year	2 Years	3 Years	4 Years	5 Years
Toh BC et al. (16)							
	Roux-En-Y Gastric Bypass	60.2%	62.1%	57.6%	50.1%	48.7%	47.7%
	Sleeve Gastrectomy	49.7%	61.2%	56.1%	47.8%	40.8%	47.3%
Lager CJ et al. (17)							
	Roux-En-Y Gastric Bypass		70.8%	69.7%			
	Sleeve Gastrectomy		55.2%	51.7%			
Khalaj et al. (18)							
	Roux-En-Y Gastric Bypass	62.7%	77.5%	80.1%			
	Sleeve Gastrectomy	61.9%	74.8%	75.0%			
Flølo TN et al. (19)							
	Sleeve Gastrectomy		76%				64%
Inge TH et al. (20)							
	Roux-En-Y Gastric Bypass						26% *Adolescent Group 29% *Adult Group
Peterli et al. (21)							
	Roux-En-Y Gastric Bypass		76.6%	77.7%	73.8%		
	Sleeve Gastrectomy		72.3%	74.7%	70.9%		
Castro MJ et al. (22)							
	Roux-En-Y Gastric Bypass		77.3%	81.9%			72.5%
	Sleeve Gastrectomy		73.6%	73.6%			68.8%

Type-2 Diabetes Mellitus

Comorbid conditions are evaluated in each patient before undergoing either RYGB or LSG procedure. Highly prevalent comorbidity in this patient population is type 2 diabetes mellitus (T2DM), defined as a hemoglobin A1C (HbA1C) greater than 6.5%, a fasting plasma glucose (FPG) greater than 126 mg/dL, or a plasma glucose greater than 200 mg/dL after a two-hour glucose tolerance test [23]. Preoperatively, the severity of this disease is recorded with consideration to medication use; patients are recorded as management without medication, use of oral medications, or use of insulin therapy [16,18,20-22,24-27]. Throughout the postoperative period, patients are reevaluated at each follow-up visit for monitoring of T2DM remission, improvement, or worsening of the disease. Remission of the disease is the converse of the earlier mentioned definition; however, most studies use the HBA1C as a marker for remission [16,18,20-22,24-27]. 

Multiple studies on the effect of bariatric surgery on T2DM have shown excellent results in the complete remission of the disease, as well as decreased medication use in those in whom remission was not achieved [16,18,20-22,24-27]. In the adolescent population, remission of diabetes was an impressive 86% after RYGB, thus resulting in consideration for RYGB as a first-line treatment for T2DM in adolescents [20]. Studies continued to follow patients up to five years postoperatively, with the majority of patients showing to be free of disease in as early as one year [20,22-26]. 

A five-year randomized, single-center study critically evaluated the effect of bariatric surgery on T2DM in comparison to intensive medical therapy alone; all periods postoperatively (12 months, three years, and five years) showed bariatric surgery with RYGB or SG in combination with intensive medical therapy, proved superior to intensive medical therapy alone for the remission of T2DM [24-26]. Further suggests that bariatric surgery may prove to be an efficacious first-line therapy for the management of obesity and comorbid conditions. Details of remission of T2DM for both RYGB and SG for the studies are listed in Table 2.

**Table 2:** Percentage of Type 2 Diabetes Mellitus Remission

	Procedure Performed	Post-Operative Follow Up and Percentage of Type 2 Diabetes Mellitus Remission				
		6 Months	1 Year	2 Years	3 Years	5 Years
Toh BC et al. (16)						
	Roux-En-Y Gastric Bypass		86.9%			
	Sleeve Gastrectomy		82.2%			
Khalaj et al. (18)						
	Roux-En-Y Gastric Bypass	56%	65.3%	63.8%		
	Sleeve Gastrectomy	73.3%	71.9%	53.3%		
Inge TH et al. (20)						
	Roux-En-Y Gastric Bypass					86% *Adolescent Group 53% *Adult Group
Peterli et al. (21)						
	Roux-En-Y Gastric Bypass				77%	
	Sleeve Gastrectomy				60%	
Castro MJ et al. (22)						
	Roux-En-Y Gastric Bypass		83.6%	83.6%	80.3%	
	Sleeve Gastrectomy		81.9%	79.5%	75.9%	
Schauer et al. (24-26)						
	Roux-En-Y Gastric Bypass		42%		38%	29%
	Sleeve Gastrectomy		37%		24%	23%
Mingrone et al. (27)						
	Roux-En-Y Gastric Bypass			75%		

Hyperlipidemia

Bariatric surgery has been shown to extend its beneficial effects on other comorbidities, including hyperlipidemia (HLD). Defined as an LDL cholesterol greater than 190mg/dL, or greater than 160mg/dL and 130mg/dL with a history of one major risk factor or two cardiovascular risk factors, respectively [28]. Secondary or “acquired” HLD with remarkable lab values of elevated LDL cholesterol or low HDL cholesterol are mainly contributed by factors such as dietary sources of cholesterol or saturated fats and are strongly correlated with central obesity [29]. To date, statin therapy has been the gold standard for HLD, with excellent results in decreasing overall cholesterol levels and decreasing cardiovascular events [28-29]. However, bariatric surgery with the RYGB or SG has also shown to reduce overall cholesterol levels, even leading to full remission of the disease at interval follow-up visits [3-6]. 

Studies following the effect of surgery on HLD have shown up to 82% of patients achieve remission of the disease at one-year follow-up after RYGB, and up to 45% of the patient following SG [18,21-22,30]. A decrease in medication use and lipid levels from baseline was also observed in those in whom full remission was not achieved [18,21-22,30]. While not the primary goal of most patients choosing to undergo bariatric surgery, the significant decrease in medication use and remission of disease observed in this patient population has been subsequently considered an additional benefit. Notably, in each study, the RYGB showed higher percentages of patients who achieved remission of HLD at all time points at follow-up [18, 21-22, 30]. Studies reporting the effect of bariatric surgery on HLD are listed in Table 3. 

**Table 3:** Percentage of Hyperlipidemia Remission

	Procedure Performed	Post-Operative Follow Up and Percentage of Hyperlipidemia Remission				
		6 Months	1 Year	2 Years	3 Years	5 Years
Khalaj et al. (18)						
	Roux-En-Y Gastric Bypass	24.7%	37.1%	29.8%		
	Sleeve Gastrectomy	25.4%	27.7%	14.2%		
Peterli et al. (21)						
	Roux-En-Y Gastric Bypass				72%	
	Sleeve Gastrectomy				44%	
Castro et al. (22)						
	Roux-En-Y Gastric Bypass		82.3%	81.2%		69.8%
	Sleeve Gastrectomy		44.9%	38.8%		26.5%
Puzziferri et al. (30)						
	Roux-En-Y Gastric Bypass			60.4%		

Hypertension 

At baseline, most patients undergoing bariatric surgery suffer from hypertension (HTN) as a comorbidity of obesity. Defined as a systolic blood pressure (SBP) of 130-139mmHg or a diastolic pressure of 80-89mmHg according to the American Heart Association [31]. Recommendations for pharmacological therapy begin at values of 140/90mmHg with a therapeutic goal of less than the aforementioned value [32]. Post-operative follow-up includes continued monitoring of blood pressure values with re-evaluation of the diagnosis at each visit. Bariatric surgery with RYGB or SG has shown to improve HTN in patients by either full remission of disease or a decrease in medication use [18,20-22,30]. At follow up, improvements in the disease have shown to occur in as soon as six months post-operatively, with percentages of patients showing remission as high as 84% [18,20-22,30]. According to studies, the RYGB has shown higher percentages of patients undergoing the procedure to achieve full remission of the disease as compared to SG [18, 20-22, 30]. Studies reporting full remission of HTN after bariatric surgery at follow up can be viewed in Table 4. 

**Table 4:** Percentage of Hypertension Remission

	Procedure Performed	Post-Operative Follow Up and Percentage of Hypertension Remission				
		6 Months	1 Year	2 Years	3 Years	5 Years
Khalaj et al. (18)						
	Roux-En-Y Gastric Bypass	46.3%	52.6%	54.7%		
	Sleeve Gastrectomy	50.4%	52.2%	39.1%		
Inge et al. (20)						
	Roux-En-Y Gastric Bypass					68% *Adolescent Group 41% *Adult Group
Peterli et al. (21)						
	Roux-En-Y Gastric Bypass				71.2%	
	Sleeve Gastrectomy				65.2%	
Castro et al. (22)						
	Roux-En-Y Gastric Bypass		84.2%	84.2%		72.6%
	Sleeve Gastrectomy		77.4%	73.6%		60.4%
Puzziferri et al. (30)						
	Roux-En-Y Gastric Bypass			38.2%		

Discussion

Treatment for obesity with bariatric surgery has shown to be efficacious for weight loss, with the added benefit of treatment for coexisting comorbidities [16-22,24-27,30]. Primary management for obesity begins with lifestyle modification, including participation in an exercise program and changes in diet. Patients in whom primary management of obesity has been unsuccessful may be considered candidates for bariatric surgery. Weight loss has shown to be the primary benefit of surgery; however, secondary benefits have followed in the way of remission or improvement of comorbid diseases [16-22,24-27,30]. According to studies, most patients undergoing bariatric surgery for treatment of obesity concurrently suffer from comorbidities such as type 2 diabetes mellitus, hypertension, and hyperlipidemia [16-22,24-27,30]. At follow-up, patients are monitored and reevaluated for ongoing weight loss or weight gain, as well as for evidence for improvement or worsening of values such as HbA1c, LDL or HDL, and systolic blood pressure. In our review, we discuss studies that follow patients post-operatively for up to five years, reporting on the outcomes of weight loss, as well as changes to baseline values of multiple comorbidities. 

At the latest follow-up of five years postoperatively, multiple studies show consistent weight loss throughout the extended postoperative period with %EWL of up to 72% (16, 19-20, 22). Furthermore, RYGB was shown to be the most impactful on the amount of weight loss (measured as %EWL) when compared to the SG at all periods of follow up, with %EWL as high as 80% [16-18, 21-22]. Such results show that surgery is an excellent alternative for weight loss management after the failure of conservative medical management. To date, the SG has gained in popularity as the most performed bariatric surgery, with percentages of EWL reaching as high as 76% and maintained weight at five years postoperatively [16,19,22]. Though the reasoning for the gain in popularity of this particular procedure is not clear, it has been proposed to be due to patients favoring a less invasive procedure with less manipulation of native anatomy. 

An essential topic of discussion in the treatment of obesity is the success of a patient in both attaining and maintaining weight loss. At first glance, many cases may appear to result in fail to initially lose weight or failure to continue to lose weight, as is shown in all patients that meet the pre-operative indications for candidacy. Bariatric surgery (either RYGB or SG) has shown dramatic weight reduction in the immediate postoperative period as well as increased loss and maintenance of weight loss several years thereafter. As a result, this can be considered a potential solution for jump-starting weight loss and health improvement in patients with obesity.

The benefits of weight loss additionally affect the treatment of comorbid conditions commonly co-existent with obesity. By way of full remission of disease or decreased medication use, bariatric surgery has exhibited a profound effect on patients with a T2DM comorbidity [16,18,20-22,24-27]. In a noteworthy trial directly comparing surgery with medical management alone for treatment of T2DM, Schauer et.al. reports that surgery in combination with medical management was superior to medical management alone for remission of T2DM [24-26]. Parameters involved in evaluating the efficacy of surgery included fasting plasma glucose and HbA1c levels, measured pre- and postoperatively. An additional study, including the pediatric population reported a higher efficacy of surgery for improvement of T2DM in the study population when directly compared to adults [20]. The study suggests the consideration of surgery as a potential first-line treatment for T2DM due to the promising results. Amongst all the studies mentioned in our review, in cases where disease remission was not observed, successful treatment of the disease was measured by a decreased medication use of oral hypoglycemic agents, metformin, or insulin therapy [16, 18, 20-22, 24-27]. 

Remission of disease was also observed in patients suffering from concurrent hyperlipidemia and hypertension [18,20-22,30]. Baseline values in multiple patients show elevated levels of LDL-cholesterol and systolic blood pressure, as reported by studies included in this review. At follow up, repeat labs show remission of disease and decreased medication use in those where remission was not achieved. Studies indicate that disease remission was maintained longitudinally, extending to the five-year postoperative follow up period [20,22]. Thus, showing an extended benefit with regard to cardiovascular disease risk factor amelioration. 

## Conclusions

With the increasing prevalence of obesity globally, an alternative management of this condition should be considered, particularly when medical management is refractory. We have discussed bariatric surgery - both LSG and RYGB procedures - as alternative (or adjunctive) therapeutic option. The beneficial effects of bariatric surgery in the obese population has been shown to extend beyond significant weight loss and leads to improvement and remission of multiple comorbidities, namely T2DM, hyperlipidemia and hypertension. As reported by studies discussed, bariatric surgery has shown to be highly efficacious in the immediate postoperative period and longitudinally across the extended follow up period. 
